# Regularized Functional Connectivity in Schizophrenia

**DOI:** 10.3389/fnhum.2022.878028

**Published:** 2022-05-11

**Authors:** Raymond Salvador, Paola Fuentes-Claramonte, María Ángeles García-León, Núria Ramiro, Joan Soler-Vidal, María Llanos Torres, Pilar Salgado-Pineda, Josep Munuera, Aristotle Voineskos, Edith Pomarol-Clotet

**Affiliations:** ^1^FIDMAG Germanes Hospitalàries Research Foundation, Barcelona, Spain; ^2^Centro de Investigación Biomédica en Red de Salud Mental, Barcelona, Spain; ^3^Department of Psychiatry, Hospital Sant Rafael, Barcelona, Spain; ^4^Benito Menni Centre Assistencial en Salut Mental, Sant Boi de Llobregat, Barcelona, Spain; ^5^Hospital Mare de Déu de la Mercé, Unitat Polivalent, Barcelona, Spain; ^6^Department of Diagnostic Imaging, Hospital Sant Joan de Déu, Barcelona, Spain; ^7^Campbell Family Mental Health Research Institute, Toronto, ON, Canada; ^8^Department of Psychiatry, University of Toronto, Toronto, ON, Canada

**Keywords:** resting state fMRI, schizophrenia, functional connectivity, ridge regression, global brain connectivity

## Abstract

Regularization may be used as an alternative to dimensionality reduction when the number of variables in a model is much larger than the number of available observations. In a recent study from our group regularized regression was employed to quantify brain functional connectivity in a sample of healthy controls using a brain parcellation and resting state fMRI images. Here regularization is applied to evaluate resting state connectivity abnormalities at the voxel level in a sample of patients with schizophrenia. Specifically, ridge regression is implemented with different degrees of regularization. Results are compared to those delivered by the weighted global brain connectivity method (GBC), which is based on averaged bivariate correlations and from the non-redundant connectivity method (NRC), a dimensionality reduction approach that applies supervised principal component regressions. Ridge regression is able to detect a larger set of abnormally connected regions than both GBC and NRC methods, including schizophrenia related connectivity reductions in fronto-medial, somatosensory and occipital structures. Due to its multivariate nature, the proposed method is much more sensitive to group abnormalities than the GBC, but it also outperforms the NRC, which is multivariate too. Voxel based regularized regression is a simple and sensitive alternative for quantifying brain functional connectivity.

## Introduction

Usage of regularization methods is obiquitous in estimating problems involving high dimensionality data (Bühlmann and Van De Geer, 2011). In MRI, where voxels are the primary unit of information representation, there may be from tens to hundreds of thousands of values characterizing an imaged brain. However, comprehensive analyses on such large data entities have been usually preceded by dimensionality reduction steps such as principal component analysis, independent component analysis, and partial least squares (Calhoun et al., 2009; McIntosh and Misic, 2013; Salvador et al., 2017) which drastically reduce the number of variables to be considered in the following analyses.

Specifically, in the field of functional connectivity one may use this newly generated subset of low dimensionality variables to fit linear models quantifying the connectivity between a single voxel and the remaining gray matter voxels of the brain (Salvador et al., 2017). These models may be considered as truly multivariate models, in contrast to other voxel level connectivity measures that are simple averages of bivariate correlations between the target voxel and the rest of voxels in the brain, as proposed by the weighted Global Brain Connectivity (GBC) method (Cole et al., 2010).

As an alternative to dimensionality reduction methods, regularization techniques allow fitting models directly to the original data by applying constraints on the parameter estimates of the fitted models (Hastie et al., 2009). This approach was recently proposed by our group and applied to analyze age and gender related connectivity patterns in a sample of healthy controls scanned at rest (Salvador et al., 2020). In this first study, mean time series from 246 regions of interest (ROIs) from the Brainnetome atlas (Fan et al., 2016) were used as input data for the regularized regressions (ridge and random forest regressions) and results obtained were compared to those delivered by the GBC, finding distinct connectivity patterns between both approximations, which were especially relevant in the age-related analyses (Salvador et al., 2020).

Such methods may also be useful in improving understanding of psychiatric disorders, such as schizophrenia. One of the most accepted ethiological hypotheses for schizophrenia is the dysconnection hypothesis (Weinberger, 1993; Friston and Frith, 1995) which states that symptoms in patients with schizophrenia arise from abnormalities in brain connectivity at several levels (Friston et al., 2016). While there are many MRI connectivity studies with significant results summarized in recent meta-analyses (e.g., Zhou et al., 2015; Giraldo-Chica and Woodward, 2017). the application and evaluation of newly developed connectivity methods remains of high interest in schizophrenia.

Here we apply one of the regularization methods used in our previous study (ridge regression) to evaluate differences in resting state connectivity between a sample of *N* = 74 patients with schizophrenia and *N* = 74 healthy controls matched for gender, age, and premorbid IQ. The association with clinical severity is also evaluated in a larger sample of *N* = 148 patients. Results are compared to those provided by the GBC method and by the non-redundant connectivity method (NRC) (Salvador et al., 2017), a dimensionality reduction approach that applies supervised principal component regressions (Bair et al., 2006; Hastie et al., 2009). We also extend the previous implementation of ridge regression by directly considering voxels instead of ROIs as inputs for the analyses.

## Materials and Methods

### Regularized Brain Connectivity

Ideally, if scanning time was not a constraint and image acquisition was carried out indefinitely, the number of recorded time observations (N) would eventually exceed the number of voxels (p) and a simple linear estimate of the association between one voxel *i* and the remaining gray matter voxels in the brain could be obtained by ordinary least squares (OLS). Under this theoretical scenario, the multiple regression equation would give the predicted time series for the target voxel (${\hat{Y}}_{i}$)

(1) $${\hat{Y}}_{i}=\beta_{0}+\beta_{1}\,Y_{1}+\ldots +\beta_{i-1}\,Y_{i-1}+\beta_{i+1}\,Y_{i+1}+\ldots +\beta_{p}\,Y_{p}$$

and a simple measure of functional connectivity for this voxel would be provided by the multiple correlation coefficient

(2) $$C\,o\,r\,\left({\hat{Y}}_{i},Y_{i}\right)$$

which quantifies the degree of similarity between the expected (${\hat{Y}}_{i}$) and observed (*Y*_*i*_) time series of voxel *i*, and which is given by the square root of the coefficient of determination (i.e., R^2^, a standard ouput from regression analyses).

However, in a real fMRI dataset the number of voxels is much larger than the number of time points (N≪p) and OLS is not feasible. This limitation may be overcome by drastically reducing the number of variables through dimensionality reduction or by considering ROIs from a brain parcellation. Here, though, there is also the alternative of using a regularization approach.

Specifically, regularization through ridge regression allows obtaining ${\hat{Y}}_{i}$ by setting a restriction on the parameter estimates of Equation 1


(3) $$\sum_{i=1}^{p}\beta_{i}^{2}<ct$$

(where *ct* stands for constant value). Such restriction may be restated as a constrained least-squares minimization regulated by a Lagrange multiplier (λ ≥ 0)


(4) $$\sum_{j=1}^{N}(y_{ij}-(\beta_{0}+\beta_{1}y_{1,j}+\ldots +\beta_{i-1}y_{i-1,j}+\beta_{i+1}y_{i+1,j}\text{ +}\ldots +\beta_{p}y_{p,j})){}^{2}+\text{λ}\sum_{i=1}^{p}{\text{β}}_{i}^{2}$$

where *y*_*i*,1_, …, *y*_*i,N*_ stand for the individual components (time points) of *Y*_*i*_.

Here, the selection of an adequate value for λ (the regularization parameter) will be important in order to achieve a good balance between bias and variance (i.e., to find a model that avoids overfitting while not being not too constrained). Furthermore, some aspects will have to be considered for the validity of ridge regression in the framework of regularized brain connectivity (RBC). Apart from the usual rescaling of variables (time series) to unit variance, λ values will have to remain fixed through all voxels and individuals, otherwise estimates of Equation 2 will not be comparable. Unfortunately, due to the lack of independence between observations in time series, the habitual cross-validation methods will not be suitable. Since there is no easy alternative to cross-validation in the current framework, we have decided to report results using a wide range of λ values (0.5, 1, 5, 10, 50, 100, and 500). All ridge regressions have been carried out with functions contained in the glmnet R library (Friedman et al., 2010).

To highlight the multivariate nature of the RBC, results obtained have been compared to those provided by simple averages of bivariate correlations. To do so, the weighted Global Brain Connectivity (GBC) method (Cole et al., 2010) has been also applied to the resting state images. Specifically, averages of absolute values of all bivariate correlations involving each specific voxel have been calculated. Results from the RBC have been also compared to connectivity estimates obtained through supervised principal component regressions (Bair et al., 2006; Hastie et al., 2009), a dimensionality reduction approach applied to quantify brain connectivity from fMRI images (Salvador et al., 2017).

### Participants

A sample of *N* = 148 patients with a diagnosis of schizophrenia according to DSM-IV criteria (i.e. excluding patients with schizoaffective and other schizophrenia related disorders) were recruited from three hospitals from Germanes Hospitalàries located in the Province of Barcelona, Spain (Hospital Bennito Meni C.A.S.M., Hospital Sant Rafael and Hospital de la Mercè). All patients but two were taking antipsychotic medication (atypical *N* = 109, typical *N* = 9, both *N* = 24, unknown *N* = 3, equivalents of Chlorpromazine: 508.82 mg (mean), 517.55 mg (SD). A second sample of *N* = 74 healthy controls were recruited from non-medical hospital staff, their relatives and acquaintances, plus independent sources in the community.

Controls reporting a history of mental illness and/or treatment with psychotropic medication or with a psychotic first-degree relative were not included. All individuals in both samples were right handed, aged 18–65, with no history of brain trauma or neurological disease, and not having shown alcohol/substance abuse in the last 12 months. All subjects gave written informed consent before participation and the study procedures were approved by the Comité de Ética de la Investigación de FIDMAG Hermanas Hospitalarias and adhered to the Declaration of Helsinki.

### Image Acquisition and Processing

Participants underwent a single MRI session in a 3.0 Tesla Philips Ingenia machine located in the Hospital Sant Joan de Déu (Barcelona, Spain) in which a resting state functional MRI (fMRI) sequence and a T1 structural image for anatomical reference were acquired. Parameters for the resting fMRI bold sequence were: TR = 2,000 ms, TE = 30 ms, flip angle = 70°, in-plane resolution = 3.5 mm × 3.5 mm, FOV = 238 mm × 245 mm, slice thickness = 3.5 mm, inter-slice gap = 0.75 mm, number of volumes = 256. Slices (32 per volume) were acquired with an interleaved order parallel to the AC-PC plane. The T1 image was acquired using a Fast Field Echo (FFE) with TR = 9.90 ms; TE = 4.60 ms; Flip angle = 8°; voxel size = 1 mm × 1 mm; slice thickness = 1 mm; slice number = 180; FOV = 240 mm). fMRI preprocessing steps included movement correction, spike scrubbing, regression of noise-independent components, non-linear normalization to the Montreal Neurological Institute space, regression of noise from ventricles and white matter, and low-frequency filtering in the 0.1–0.02 Hz interval (Salvador et al., 2017). Specifically, for the regression of noise-independent components, individual independent component analyses were previously run with Melodic, a module included in FSL (Smith et al., 2004) and those components showing clear noise patterns (most frequently edge effects due to movement) were selected. Time series of the selected components were regressed out from the time series of each voxel, and residuals were kept as the denoised time series.

Prior to the calculation of the RBC, GBC, and NRC maps a common gray matter mask was applied to the normalized fMRI images. Then, fMRI volumes were resampled to a 4 mm × 4 mm × 4 mm voxel in order to reduce computational costs. Finally, values from the individual RBC, GBC, and NRC maps where Fisher transformed before carrying out the group analyses.

### Group Comparisons and Group Level Regressions

A subsample of *N* = 74 patients matched for gender, age and premorbid IQ to the sample of *N* = 74 controls was selected from the original sample of patients before carrying out group comparisons in RBC, GBC, and NRC. Premorbid IQ was estimated with the Word Accentuation Test (Del Ser et al., 1997). Positive and Negative Syndrome Scale (PANSS) scores (Kay et al., 1987) were used to calculate values of the three Liddle Syndromes (Positive, Negative, and Disorganization) (Liddle, 1987) for all *N* = 148 patients, and regressions between RBC, GBC, and NRC and clinical severity as measured by the three Syndromes were performed in this larger sample. In the regression analyses, age, gender, type of antipsychotic medication (atypical and/or typical) and dose of medication (equivalents of Chlorpromazine) were considered as nuisance covariates. In all analyses statistical significance was derived from permutation tests carried out using the randomize function included in FSL (Smith et al., 2004) and applying the Threshold-Free Cluster Enhancement (TFCE) method.

## Results

A summary of clinical and demographical data for the initial sample of patients with schizophrenia and for the matched samples of patients and healthy controls is provided in Table 1.

**TABLE 1:** Summary of demographic and clinical data.

	All patients (*N* = 148)	Matched patients (*N* = 74)	Matched controls (*N* = 74)	Statistical tests and *p*-values
Gender	97M/51F	43M/31F	43M/31F	χ^2^ = 0, *p*val = 1.00
Age	42.37 (10.97)	41.12 (11.83)	38.09 (13.65)	t = –1.44, *p*val = 0.15
Premorbid IQ	21.15 (4.79)	23.14 (4.34)	23.12 (4.49)	*t* = –0.02, *p*val = 0.98
Positive Syndrome	10.41 (4.61)	10.93 (5.15)		
Negative Syndrome	14.02 (6.60)	12.86 (6.41)		
Disorganization Syndrome	7.01 (2.43)	6.74 (2.27)		

*Absolute frequencies for gender, and mean and standard deviations for age, Premorbid IQ (as estimated by the Word Accentuation Test) and the three Liddle Syndromes extracted from the PANSS scale are reported together with results from statistical tests comparing values for the matched samples of patients and healthy controls.*

### Group Analyses

**FIGURE 1:**
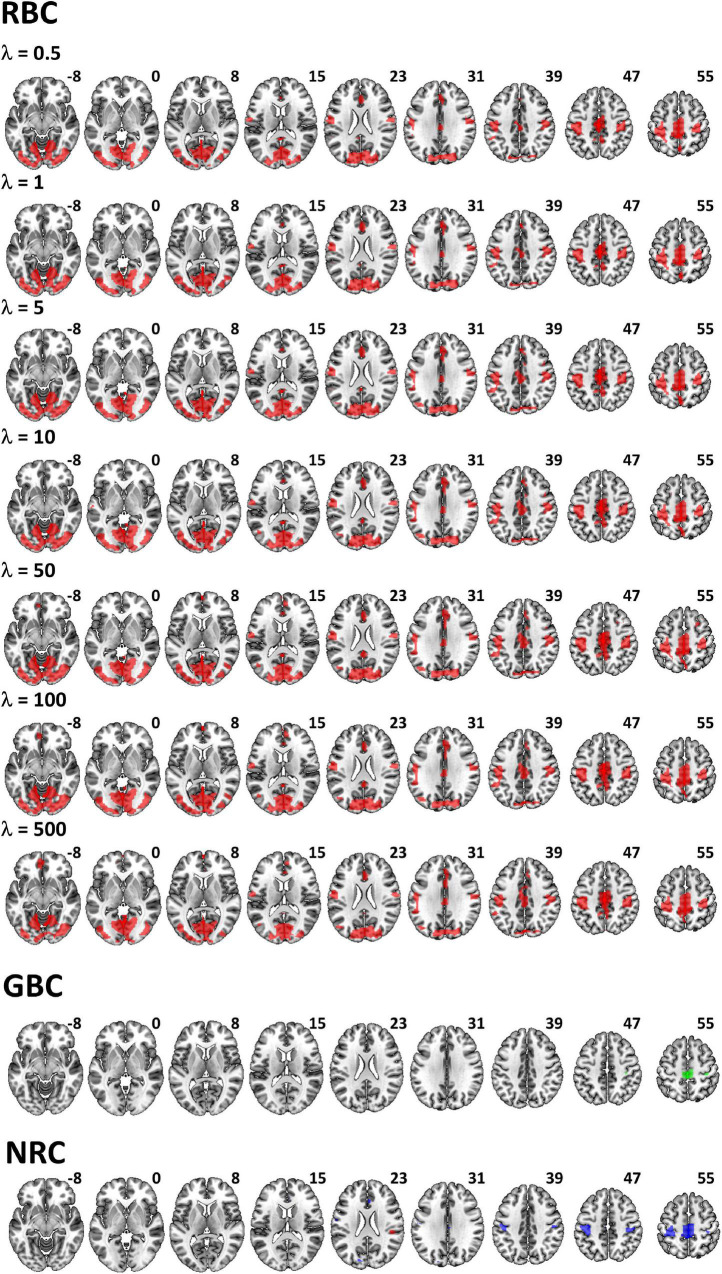
Brain areas with significant differences in RBC, GBC and NRC between patients and healthy controls. RBC results are given for the different regularization values (λ values) applied in the ridge regressions. While for the RBC (red) and GBC (green), comparisons only included reductions in connectivity, significant disorder related reductions (blue) and increases (red) were observed with the NRC (although the later were of much smaller extent).

**FIGURE 2:**
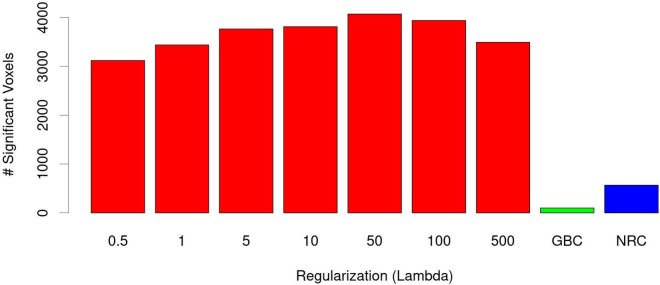
Number of voxels with significant reductions in RBC connectivity in patients as a function of λ (red bars). Most extensive abnormalities were observed with λ = 50. In all cases these were much larger than reductions observed with the GBC (green) and the NRC (blue).

When RBC maps from patients were compared to those of healthy controls only areas of significant reductions in connectivity were found. As shown in Figure 1, this pattern was consistent through all regularization values. Tests performed with λ = 50 produced the most extensive differences (Figure 2) including bilateral connectivity reductions in the supplementary motor area, paracentral lobule, precentral and postcentral gyri, precuneus, dorsal and anterior cingulate, ventromedial and right dorsolateral prefrontal cortex, cuneus, calcarine, lingual, lateral occipital areas and parts of the cerebellum.

All λ values applied led to much more widespread patterns of differences than those provided by both the GBC and NRC methods, which were mainly restricted to connectivity reductions in clusters of moderate size in the supplementary motor area / paracentral lobule and postcentral gyrus (Figure 1). As shown in Figure 2, though, reductions in the NRC were clearly larger than those observed with the GBC. A cluster of increased connectivity in patients was also observed in the right Rolandic operculum (Figure 1).

### Clinical Covariates

**FIGURE 3:**
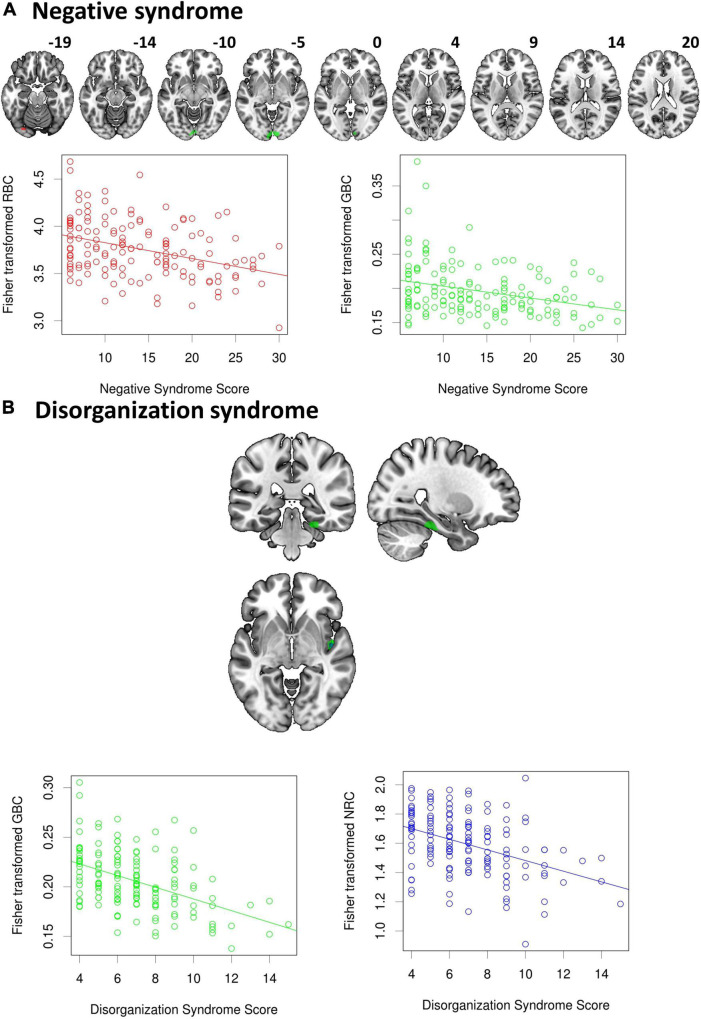
Significant associations between scores from the Liddle syndromes and connectivity levels. While **(A)** a negative relation was observed between the Negative syndrome and both the RBC and GBC in occipital areas, **(B)** the Disorganization syndrome was negatively correlated with the GBC and the NRC, but the latter only involved a very small cluster located in the right posterior insula. No association was found with positive syndrome scores.

When RBC maps were correlated with scores of the three Liddle Syndromes, a decreasing pattern of connectivity was observed for the Negative Syndrome (Figure 3A). This negative association involved a cluster located in the left occipital cortex (clusters shown in Figure 3 are based on analyses using λ = 50, which led to most extensive differences in the group comparison). Another cluster of negative association with the Negative Syndrome was observed for the GBC, which was also in the occipital cortex, although with a more medial position. The GBC also showed two clusters of negative association with the Disorganization Syndrome, one of them located in the right fusiform area, and the other in the right posterior insula (Figure 3B). The later was also the site for the only significant cluster observed with the NRC, which was of small size and was also negatively correlated with the Disorganization Syndrome. No significant associations were found between any of the three connectivity measures and the Positive Syndrome.

## Discussion

As shown by the results, the RBC is much more sensitive to group differences between patients with schizophrenia and healthy controls than the GBC. The RBC and the GBC are looking at different aspects of brain connectivity. While the GBC only accounts for net differences in bivariate correlations, the RBC is a truly multivariate measure and, consequently, it may be much more sensitive to local changes in brain connectivity (changes that may go unnoticed after the averaging operation carried out by the GBC). Still, as shown by the associations found with the Liddle syndromes, the GBC may convey relevant information. Unexpectedly, the NRC, a method that is also multivariate, did not perform as sensitively as the RBC in the group comparisons, suggesting that regularization may be a better option for quantifying functional connectivity than dimensionality reduction. However, this statement may require testing through exhaustive analyses.

Results found here are, to some extent, similar to those reported in previous studies using similar methods. In a prior study on patients with schizophrenia using the GBC (Cole et al., 2011) the authors only found reduced frontal connectivity in the primary (non seed based) analyses. A similar pattern of frontal connectivity reduction was observed in another study using GBC in schizophrenia (Yang et al., 2014) although this pattern was reversed when the global brain signal was ignored in the pre-processing phase [regrettably, pre-processing steps may have a significant effect on resting state results (Caballero-Gaudes and Reynolds, 2017)]. Using global Functional Connectivity Density mapping (gFCD), an approach similar to GBC, based on the number of correlations above a certain threshold connecting each voxel (Tomasi and Volkow, 2010), Zhuo et al. (2017) also reported a similar pattern of disconnections in patients with schizophrenia, which included reductions in connectivity in postcentral gyri, occipital cortex, temporo-occipital conjunction, and inferior parietal lobule, although these authors also found increases in subcortical structures. On the other hand, the only study using NRC in patients with schizophrenia, which was conducted at our institution in another sample of patients and healthy controls, revealed a similar pattern of disconnection in somatosensory and occipital regions (Salvador et al., 2017), although a small cluster of increased connectivity was also found in the Caudate and no significant differences were reported for the GBC.

The dominance of reduced connectivity in these results agrees with recent meta-analyses on schizophrenia (Dong et al., 2018; Doucet et al., 2020). However, the reported abnormalities may not be restricted to schizophrenia since, although with different intensities, they have been also described in other psychotic disorders such as the bipolar disorder or major depression disorder (Xia et al., 2019). Still, they seem to be more prominent in schizophrenia (Li et al., 2021).

As previously explained in the methods, some aspects should be kept in mind in order to avoid biases and inconsistencies when applying the RBC. Most importantly λ should be kept fixed through all analyses. The selection of its value is also of relevance, as a good trade-off between variance and bias should be achieved. Unfortunately, there is no a *priori* rule for the selection of λ as its optimal value will depend on many sample specific aspects such as the number of voxels or the degree of autocorrelation and length of time series. Even so, as shown by the stability of results reported in our study using a wide range of λ values, it may be concluded that λ selection, although relevant, it does not require high accuracy.

In summary, in this study we prove that regularization, and specifically ridge regression, may be a feasible alternative to dimensionality reduction for multivariate functional connectivity estimation, even if applied at the voxel level. The regularized brain connectivity approach is able to detect a much extended set of abnormally connected regions than those detected by the global brain connectivity and the non-redundant brain connectivity methods when it is applied to a sample of patients with schizophrenia.

## Data Availability Statement

The data analyzed in this study is subject to the following licenses/restrictions: The data supporting the conclusions of this article will be made available by the authors upon sensible request. Requests to access these datasets should be directed to RS, rsalvador@fidmag.com.

## Ethics Statement

The studies involving human participants were reviewed and approved by Comité de Ética de la Investigación Clínica (CEIC-CEI) FIDMAG Hermanas Hospitalarias. The patients/participants provided their written informed consent to participate in this study.

## Author Contributions

RS, PF-C, AV, and EP-C designed the study. PF-C, MG-L, NR, JS-V, MT, JM, PS-P, RS, and EP-C collected the data. RS, AV, and EP-C analyzed the data. RS and EP-C wrote the manuscript. All authors reviewed the written text.

## Conflict of Interest

The authors declare that the research was conducted in the absence of any commercial or financial relationships that could be construed as a potential conflict of interest.

## Publisher’s Note

All claims expressed in this article are solely those of the authors and do not necessarily represent those of their affiliated organizations, or those of the publisher, the editors and the reviewers. Any product that may be evaluated in this article, or claim that may be made by its manufacturer, is not guaranteed or endorsed by the publisher.
